# Cellular Tight Junctions Prevent Effective *Campylobacter jejuni* Invasion and Inflammatory Barrier Disruption Promoting Bacterial Invasion from Lateral Membrane in Polarized Intestinal Epithelial Cells

**DOI:** 10.3389/fcimb.2018.00015

**Published:** 2018-01-30

**Authors:** Sho Hatayama, Takaaki Shimohata, Sachie Amano, Junko Kido, Anh Q. Nguyen, Yuri Sato, Yuna Kanda, Aya Tentaku, Shiho Fukushima, Mutsumi Nakahashi, Takashi Uebanso, Kazuaki Mawatari, Akira Takahashi

**Affiliations:** ^1^Department of Preventive Environment and Nutrition, Institute of Biomedical Science, Tokushima University, Tokushima, Japan; ^2^Graduate School of Technology, Industrial and Social Sciences, Tokushima University, Tokushima, Japan

**Keywords:** *Campylobacter jejuni*, Tight Junctions (TJs), lipid rafts, cell invasion, lateral part of epithelial cells

## Abstract

*Campylobacter jejuni* invasion is closely related to *C. jejuni* pathogenicity. The intestinal epithelium contains polarized epithelial cells that form tight junctions (TJs) to provide a physical barrier against bacterial invasion. Previous studies indicated that *C. jejuni* invasion of non-polarized cells involves several cellular features, including lipid rafts. However, the dynamics of *C. jejuni* invasion of polarized epithelial cells are not fully understood. Here we investigated the interaction between *C. jejuni* invasion and TJ formation to characterize the mechanism of *C. jejuni* invasion in polarized epithelial cells. In contrast to non-polarized epithelial cells, *C. jejuni* invasion was not affected by depletion of lipid rafts in polarized epithelial cells. However, depletion of lipid rafts significantly decreased *C. jejuni* invasion in TJ disrupted cells or basolateral infection and repair of cellular TJs suppressed lipid raft-mediated *C. jejuni* invasion in polarized epithelial cells. In addition, pro-inflammatory cytokine, TNF-α treatment that induce TJ disruption promote *C. jejuni* invasion and lipid rafts depletion significantly reduced *C. jejuni* invasion in TNF-α treated cells. These data demonstrated that TJs prevent *C. jejuni* invasion from the lateral side of epithelial cells, where they play a main part in bacterial invasion and suggest that *C. jejuni* invasion could be increased in inflammatory condition. Therefore, maintenance of TJs integrity should be considered important in the development of novel therapies for *C. jejuni* infection.

## Introduction

*Campylobacter jejuni* is a Gram-negative, spiral-shaped, microaerophilic bacterium that is found in birds and domestic animals. *C. jejuni* causes human bacterial food-borne diseases worldwide, and clinical symptoms are manifested as intestinal inflammation, abdominal pain, and diarrhea (Young et al., 2007). Several studies reported that *C. jejuni* can adhere to and invade epithelial cells in an infection process that induces secretion of the pro-inflammatory cytokine interleukin (IL)-8 by intestinal epithelial cells (Konkel and Jones, 1989; Hickey et al., 1999). IL-8 production recruit neutrophils to the infection site and subsequently host inflammatory responses to *C. jejuni* infection. Moreover, the mutant *C. jejuni* strains lacking invasion activity had attenuated inflammatory responses and several diarrhea symptoms in experimental animal models (Yao et al., 1997). Together these findings indicate that bacterial invasion into host intestinal epithelial cells plays a critical role in *C. jejuni* pathogenicity.

Previous studies identified several bacterial and host cellular factors involved in *C. jejuni* adherence and invasion. An extracellular matrix protein, fibronectin, is one of the characterized host cellular factors which interacts with *C. jejuni* adherence and some reports indicated that *C. jejuni* binding factor, CadF and FlpA protein, were involved in maximal adherence for the host cell (Monteville et al., 2003; Konkel et al., 2010). Moreover, a surface-exposed bacterial lipoprotein, JlpA, has also been reported as a key adherence factor for *C. jejuni* and it bound HSP-90α, a heat shock protein in host cells (Jin et al., 2001, 2003). In addition, the bacterial ABC transporter component PEB1 and an autotransporter protein CapA also mediated both adherence and invasion in host epithelial cells (Pei et al., 1998; Ashgar et al., 2007). Bipolar flagella or a major flagellin component FlaA had an important role in both motility of *C. jejuni* and bacterial invasion into host cells (Wassenaar et al., 1991). In addition to these function, *C. jejuni* flagella secretion system, similar with a Type III secretion system, was required for maximal cell invasion (Konkel et al., 1999; Christensen et al., 2009; Samuelson et al., 2013). Meanwhile, in the *C. jejuni* trafficking mechanisms, lipid rafts, which are well-known as cholesterol- and sphingolipid-rich plasma membrane microdomain, were essential for *C. jejuni* entry via caveolae-mediated endocytosis pathway (Wooldridge et al., 1996). Following to endocytosis, microfilaments and microtubules were required for *C. jejuni* translocation (Oelschlaeger et al., 1993; Biswas et al., 2003). Importantly, the cytotoxicity in *C. jejuni* infection was closely related with bacterial invasion ability and is independent of major virulence factor, such as cytoletal distending toxin (CDT) (Kalischuk et al., 2007).

The detail mechanisms of *C. jejuni* invasion have been investigated in non-polarized epithelial cells. For example, some earlier reports revealed that *C. jejuni* utilized the host cell scaffolding protein and signaling cascade to invade into host cells, including integrin, epidermal growth factor receptor (EGFR), focal adhesion kinase (FAK), and paxillin (Monteville et al., 2003; Boehm et al., 2011; Eucker and Konkel, 2012). In addition, Rho small GTPase Rac1 and Cdc42 activation also take part in *C. jejuni* entry (Krause-Gruszczynska et al., 2007). Those findings came from non-polarized epithelial cells using studies. In contrast, there were few report to examine the molecular mechanism of *C. jejuni* invasion in polarized epithelial cells. Few studies reported that *C. jejuni* invasion was attenuated by the host barrier function and this attenuation of *C. jejuni* invasion was mainly mediated by the apical junctional complexes termed tight junctions (TJs) (Beltinger et al., 2008). On the other hand, other studies reported that *C. jejuni* disrupted TJs and its disruption of TJs promoted *C. jejuni* invasion into intestinal epithelial cells from the basolateral regions of host cells (Monteville and Konkel, 2002; Chen et al., 2006; van Alphen et al., 2008; Bouwman et al., 2013). Despite some findings of the association between TJs and the invasion in non-polarized epithelial cell, the bacterial invasion mechanisms were poorly understood in polarized epithelial cells. We hypothesized that *C. jejuni* encountering host cellular factors locates in lateral or basolateral part, but TJs may prevent the *C. jejuni* encounter with host cellular factors in polarized epithelial cells.

In this study, we examined whether TJ impedes host trafficking mechanisms and contributes to *C. jejuni* invasion using polarized epithelial cells with both intact or TJ disrupted cell. We found difference in *C. jejuni* invasion was different between polarized and non-polarized epithelial cells, and *C. jejuni* invasion process in TJ-disrupted or TJ-unformed polarized cells were similar to that in non-polarized epithelial cells. In addition, calcium switch assay revealed that *C. jejuni* invasion was tightly regulated by TJ function. Furthermore, pro-inflammatory cytokine-mediated TJ disruption also increased the *C. jejuni* invasion process in polarized epithelial cells, and the date indicated that TJ disruption induced by both pharmacologic and physiological factor was affective in *C. jejuni* invasion. Here we firstly indicated the *C. jejuni* invasion process was similar to intestinal inflammatory state. Our data suggested that TJs interfered with *C. jejuni* invasion at cellular lateral membranes and physiological TJ disruption such as inflammatory response could facilitate *C. jejuni* invasion in polarized epithelial cells. Together these findings could help elucidate *C. jejuni* invasion mechanisms into host intestinal tissues.

## Materials and methods

### Bacterial strains and culture conditions

*Campylobacter jejuni* strains ATCC 700819 (NCTC11168) were obtained from American Type Culture Collection (ATCC). The bacteria were grown in Muller Hinton Broth (DIFCO) under a microaerobic atmosphere (5% O_2_, 10% CO_2_, 85%N_2_) at 37°C for 48 h. The bacteria were then diluted into fresh MH and cultured under a microaerobic atmosphere at 37°C for an additional 36 h. *Salmonella enterica* serovar enteritidis (*S*. Enteritidis) was cultured in Luria-Bertani (LB) medium at 37°C with shaking.

### Cell culture

The non-polarized cell lines HeLa, INT407 and polarized cell lines Caco-2 cells were cultured in Dulbecco's modified Eagle's medium (DMEM), high-glucose (Sigma-Aldrich) supplemented with 10% fetal bovine serum (FBS; GIBCO) and 50 μg/ml gentamycin (Sigma-Aldrich). The culture medium was changed every 4 days in Caco-2 cells. The culture medium was changed every 4 days. Polarized cell lines T-84 cells were cultured in Dulbecco's modified Eagle's medium nutrient mixture F-12 HAM (DMEM/F-12, 1:1; Sigma-Aldrich) supplemented with 10% FBS and 50 μg/ml gentamycin. The culture medium was changed every 2 days. All cells were incubated in 37°C in a humidified atmosphere containing 5% CO_2_.

### Regent and antibody

Ethylene Glycol Tetra-acetic Acid (EGTA) was purchase from Nacalai Tesque. Rhodamine Phalloidin was purchase from Molecular Probes. Methyl-β-cyclodextrin (MβCD), water soluble-cholesterol and FITC-dextran (4 kDa, 10 kDa) were purchase from Sigma-Aldrich. U18666A and Tumor necrosis factor-α (TNF-α) were purchase from Wako. Antibodies to the following were diluted in 3% BSA and used for Immunofluorescence staining ZO-1 (1:200; BD Bioscience), Alexa Fluor 568 (1:200, Molecular Probes).

### Infection protocols

Bacteria were harvested by centrifugation at the 3,000 rpm for 15 min in *C. jejuni* infection or 12000 rpm for 3 min in *S*. Enteritidis infection and supernatant was removed. The bacteria were washed by phosphate buffered saline (PBS; 137 mM NaCl, 8.1 mM anhydrous

Na2HPO4, 2.68 mM KCl, 1.47 mM KH2PO4, pH 7.4), centrifuged and resuspended in PBS. The bacteria concentration was adjusted to an optical density of 600 nm (OD_600_) of 1.0 by PBS. Before infection, the culture medium was removed and replaced with fresh DMEM medium without supplements. Cells were infected at a multiplicity of infection (MOI) of 100–200:1 in *C. jejuni* infection or 10–20:1 in *S*. Enteritidis infection. Infected cells were incubated at 37°C in 5% CO_2_.

### Invasion and adhesion assay

INT407 and HeLa cells were seeded at a density of 1.0 × 10^5^/well in 24-well plate and cultured for 3 days. Polarized Caco-2 and T84 cells were seeded at a density of 7.5 × 10^4^/well and 2.5 × 10^5^/well in 24-well plate and cultured for 7 days. For basolateral infection, Caco-2 cells were seeded at a density of 1.0 × 10^5^/well in inverted 0.33 cm^2^ transwell insert containing 3.0 μm pores (Corning). After 6–9 h incubation, the cell seeded inserts were re-inverted into DMEM high glucose and cultured for 1 week. The cell culture medium was replenished every 2 days and cells were used for invasion experiments.

For invasion assay, cells were infected with *C. jejuni* for 6 h or *S*. Enteritidis for 1 h. After infection, the cells were incubated with gentamycin containing DMEM (100 or 500 μg/ml for *C. jejuni* and *S. enteritidis*, respectively) for 2 h to kill extracellular bacteria. After incubation, the cells were washed with PBS and lysed with 1% Triton-X in PBS for 5 min at 37°C. For adhesion experiments, cells were infected with bacteria at a MOI 20–30:1 for 1 h. After infection, the cells were washed five times with PBS and lysed with 0.1% Triton-X in PBS for 5 min at 37°C. The diluted cell lysates were plated on MH agar plates and incubated for 48 h under a microaerobic atmosphere. The number of intracellular bacteria was determined by counting colony forming units (CFU) and the values were normalized with respect to the protein concentration of the individual cells. Protein concentration was measured by using a BCA Protein Assay Kit (Thermo Fisher) according to the manufacturer's instructions.

### Disruption of tight junctions (TJs)

To disrupt cellular TJs, differentiated Caco-2 or T-84 cells were incubated with 4 mM EGTA for 20 min or 20 ng/ml TNF-α for 48 h. After treatment, the cells were then washed one time with fresh free DMEM and used for infection.

### Inhibitor study for lipid raft-mediated bacterial invasion

To disrupt lipid rafts, cells were treated with various concentrations of MβCD (1, 5, 10 mM), which removes cholesterol from the plasma cell membrane, and U18666A (7.5, 15, 30 μM) to block intracellular cholesterol trafficking and biosynthesis for 1 h prior to infection. The concentration of each inhibitor used in this study was determined by the previous publication of Elmi et al. (2012) and Konkel et al. (2013) for MβCD or Chen et al. (1993) and Field et al. (2008). for U18666A. respectively. About U18666A, we used twice higher concentration in this study compare to the previous reports because of the incubation time difference. We checked host cell viability by protein content in each experiment. In cholesterol complement assays, MβCD treated cells were incubated with water-soluble cholesterol (150 μg/ml) containing DMEM for 1 h. After treatment, the cells were then washed one time with fresh free DMEM and used for infection. To evaluate the effect of these treatment on cells, intracellular cholesterol levels were measured with a cholesterol assay kit (Wako) according to the manufacturer's instructions.

### Immunofluorescence staining

Caco-2 cells were seeded on glass cover slips at a density of 3 × 10^5^/dish and were infected for 6 h with bacteria that had been previously incubated with the CFDA SE cell trace kit reagent (Molecular Probes) for 1 h in accordance with the manufacturer's instructions. After infection, the cells were washed with PBS and fixed with 4% paraformaldehyde in PBS at room temperature for 10 min. After washing three times with PBS, the cells were permeabilized with 0.2% Triton-X-PBS for 10 min and then treated with 3% BSA in PBS for 1 h before washing 3 times with PBS. The cells were incubated with primary antibody overnight at 4°C and washed 3 times with PBS before incubation with a secondary antibody conjugated with Alexa Fluor 568 at room temperature for 60 min and washing 3 times with PBS. For F-actin staining, cells were incubated with Rhodamine Phalloidin diluted in PBS (1: 100) at room temperature for 60 min and washing 3 times with PBS.

### Transepithelial electrical resistance measurement and calcium switch assay

Caco-2 cells were seeded at a density of 1.0–2.0 × 10^5^/well on 0.33 cm^2^ transwells for TER assessment. TER was measured using an EVOM epithelial Volt-Ohm meter (World Precision Instruments) in accordance with the manufacturer's instructions. For calcium switch assays, Caco-2 cells cultured 1 week were treated with EGTA as described above and were then washed with fresh medium and re-introduced into normal medium containing calcium. Cells were incubated at 37°C for indicated time and TER values was measured and invasion assay was performed.

### Uptake of FITC-dextran

Caco-2 cells were seeded at a at a density of 7.5 × 10^4^/well and cultured for 1 week in 24-well plates following treatment with EGTA or MβCD as described above. After treatment, cells were washed with HEPES buffer (10 mM HEPES, 145 mM NaCl, 10 mM glucose, 5 mM KCl, 1 mM MgCl_2_, 1 mM CaCl_2_) and incubated with HEPES buffer containing either 0.2 mg/ml 4 kDa or 10 kDa FITC-dextran for 2 h. After incubation, the cells were washed five times with HEPES buffer and lysed with 1% Triton-X. Cell lysates were centrifuged at the 12,000 rpm for 10 min and were collected supernatant. Fluorescence in supernatant was measured by using fluorescence microplate reader at excitation and emission wavelengths of 492 and 520 nm, respectively.

### Statistical analyses

In all case, statistical analysis was performed by using student's *t*-test for pair date. Date from 3 independent experiments were evaluated. All tests were one-tailed.

## Results

### Lipid raft disruption does not affect *C. jejuni* invasion in polarized epithelial cells

**Figure 1:**
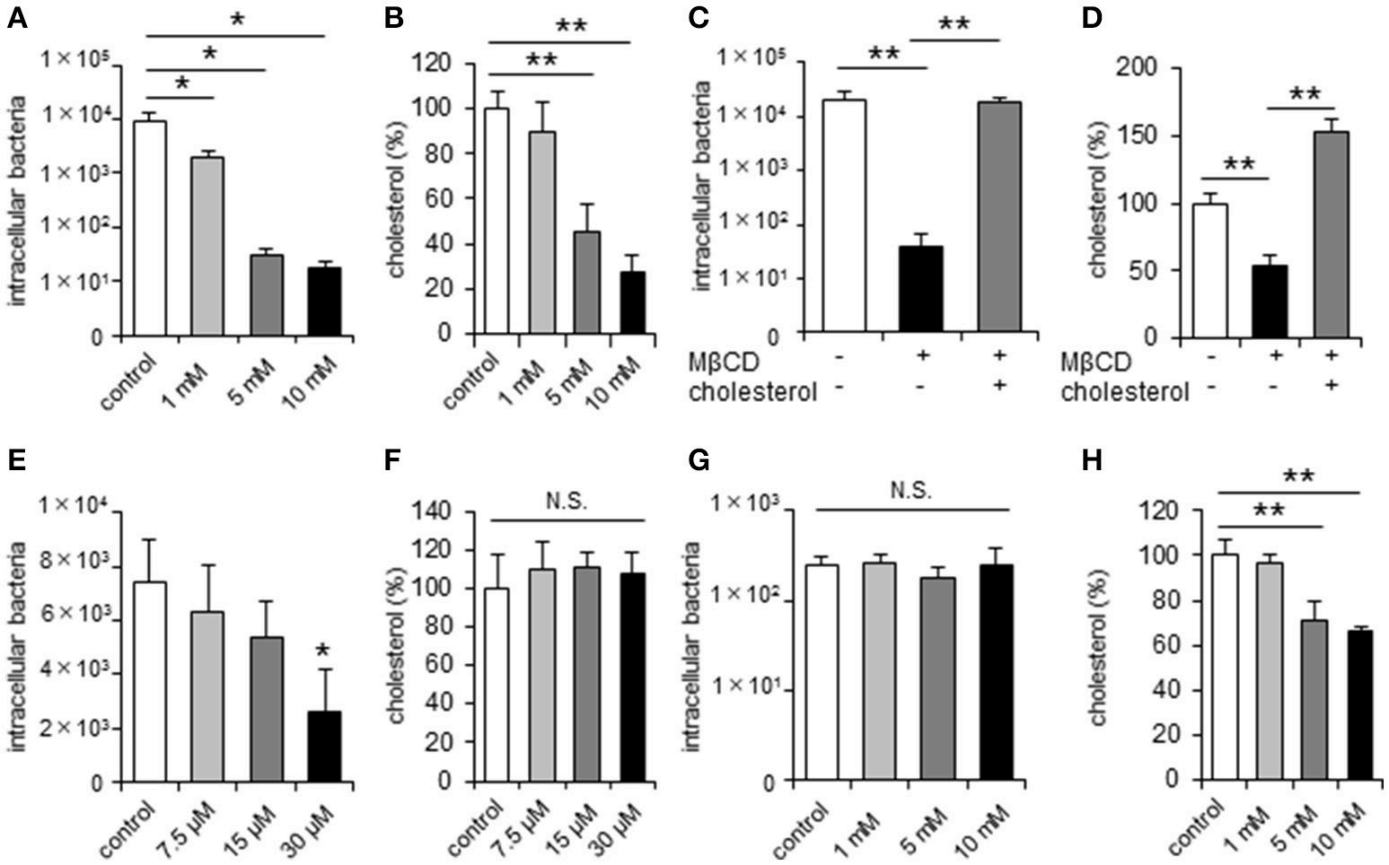
*C. jejuni* invasion is unchanged by disruption of lipid rafts in polarized epithelial cells. Non-polarized epithelial cells (INT407 cells) and polarized epithelial cells (Caco-2 cells) were pretreated with the indicated inhibitor and infected with *C. jejuni* for 6 h. The number of intracellular bacteria was measured using a gentamycin protection assay **(A,C,E,G)**. The level of intracellular cholesterol was measured using a cholesterol assay kit **(B,D,F,H)**. **(A,B)** INT407 cells treated with 1, 5, 10 mM MβCD for 1 h, **(C,D)** INT407 cells pretreated with 10 mM MβCD for 1 h or MβCD-treated cells subsequently supplemented with 150 μg/μl cholesterol for 1 h prior to infection. **(E,F)** INT407 cells pretreated with 7.5, 15, 30 μM U18666A for 1 h. **(G,H)** Caco-2 cells pre-treated with 1, 5, 10 mM MβCD for 1 h. Results are shown as the mean ± SD; *n* = 4-6. Significant difference from the control or MβCD treated group are shown: N.S.; not significant; ^*^P < 0.05; ^**^P < 0.01.

Previous studies indicated that *C. jejuni* can invade host cells via lipid raft-mediated endocytosis in non-polarized epithelial cells (Wooldridge et al., 1996). Here we found that treatment of the non-polarized cell lines INT407 and HeLa (Figures 1A,B, Supplementary Figure 1) with methyl-β-cyclodextrin (MβCD), a potent lipid raft-mediated endocytosis inhibitor that removes cellular cholesterol, significantly and dose-dependently decreased both cellular cholesterol content and *C. jejuni* invasion. This reduction could be recovered by cholesterol supplementation (Figures 1C,D, Supplementary Figure 1). To further confirm the contribution of lipid rafts to *C. jejuni* invasion, we analyzed the effect of U18666A, an inhibitor of intracellular cholesterol trafficking, on *C. jejuni* invasion. U18666A treatment of INT407 cells significantly decreased *C. jejuni* invasion without affecting the cholesterol content (Figures 1E,F). Together these results indicated that cell surface cholesterol and lipid rafts are essential for *C. jejuni* invasion of non-polarized epithelial cells. Next, we investigated the dynamics of *C. jejuni* invasion in polarized epithelial cells using Caco-2 and T-84 cells. In contrast to non-polarized epithelial cells, MβCD treatment of polarized Caco-2 and T-84 cells did not affect *C. jejuni* invasion, despite the reduction in intracellular cholesterol content (Figures 1G,H, Supplementary Figure 1), suggesting that the dynamics of *C. jejuni* invasion might differ between non-polarized and polarized epithelial cells.

### Lipid raft disruption decreases *C. jejuni* invasion in TJ unformed cells and basolateral surface infection of polarized epithelial cells

**Figure 2:**
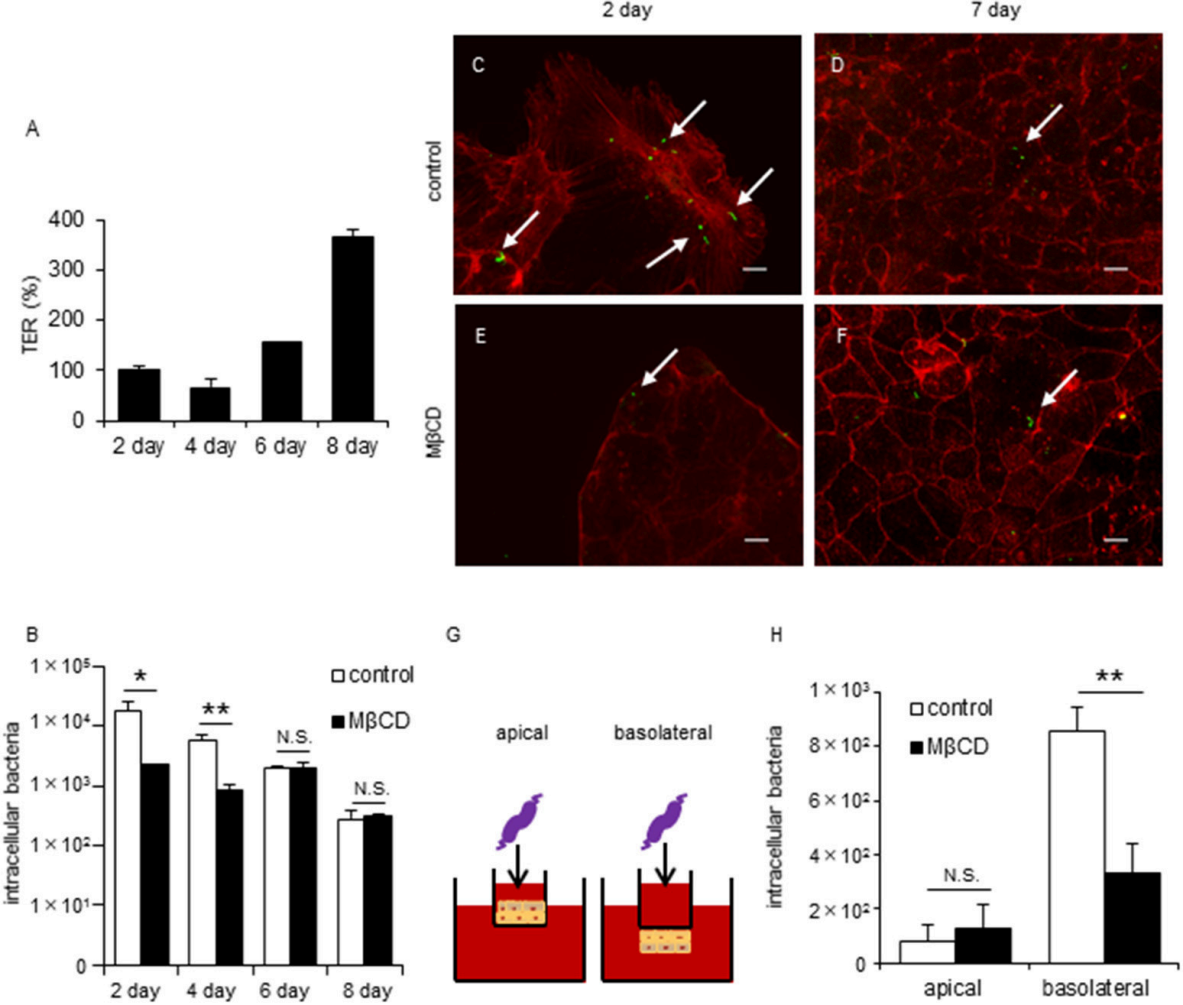
Disruption of lipid rafts reduces *C. jejuni* invasion of TJ unformed cells or infection of basolateral surfaces in polarized epithelial cells. Caco-2 cells were infected with *C. jejuni* for 6 h and the number of intracellular bacteria was measured by a gentamycin protection assay **(B,H)**. **(A)** TER values were measured as a marker of TJ formation at 2, 4, 6, and 8 days post-seeding of Caco-2 cells cultured on transwells. TER values were calculated as the percentage of Day 2 post-seeding TER values. **(B)** Caco-2 cells cultured for 2, 4, 6, and 8 days following pre-treatment with 1-10 mM MβCD (black bar) or medium only (white bar) for 1 h. **(C–F)** Caco-2 cells cultured for 2 days **(C,E)** or 7 days **(D,F)** after pre-treatment with 1–10 mM MβCD for 1 h and subsequent infection with *C. jejuni* for 6 h. After infection, the cells were fixed, permeabilized and stained for F-actin (red). *C. jejuni* (green) was visualized using a CFDA SE cell tracer kit. Arrows indicate intracellular *C. jejuni*. Untreated control cells **(C,D)** and cells treated with MβCD alone **(E,F)** are also shown. Scale bar = 10 μm. **(G,H)** Caco-2 cells cultured in normal (apical) or inverted fashion (basolateral) on transwell inserts after pre-treatment with 10 mM MβCD for 1 h. Results are shown as the mean ± SD; *n* = 4. Significant difference from the control group are shown: N.S.; not significant; ^*^P < 0.05; ^**^P < 0.01.

To investigate differences in *C. jejuni* invasion dynamics between non-polarized and polarized epithelial cells, we focused on tight junctions (TJs) formation in polarized epithelial cells. Cell polarization is established by TJ formation that separates the plasma membrane into apical and basolateral regions. Therefore, we performed invasion assays at different stages of cell culture to evaluate the relationship between TJ formation and *C. jejuni* invasion process. TJ formation of Caco-2 cells was assessed by measuring Trans Epithelial Resistance (TER) as previously described (Goyer et al., 2016) and this result showed significantly increase of TER values on day 6 post-seeding (Figure 2A), suggesting that mature TJs are present at this time point. As the TER values increased, the amount of *C. jejuni* invasion decreased. Interestingly, MβCD treatment significantly decreased *C. jejuni* invasion only in the short term (day 2 or 4 post-seeding) in cultured Caco-2 cells (Figure 2B). Similar results were seen for Caco-2 cells in a fluorescence microscopy assay using a CFDA SE cell tracer kit (Figures 2C–F). Based on these data, we hypothesized that the lateral or basolateral part of cells may contribute to *C. jejuni* invasion dynamics and *C. jejuni* susceptibility. Thus, we next performed an invasion assay using inverted-transwell inserts (Figure 2G). *C. jejuni* invasion was promoted in the basolateral region of cells, and MβCD treatment significantly reduced *C. jejuni* invasion only in the basolateral region (Figure 2H). These results suggested that basolateral region was critical for bacterial invasion in TJ formed cells, and that TJs provide the interface for the interaction between lipid rafts and invading *C. jejuni*.

### TJ disruption is closely related to lipid raft-mediated *C. jejuni* invasion in polarized epithelial cells

**Figure 3:**
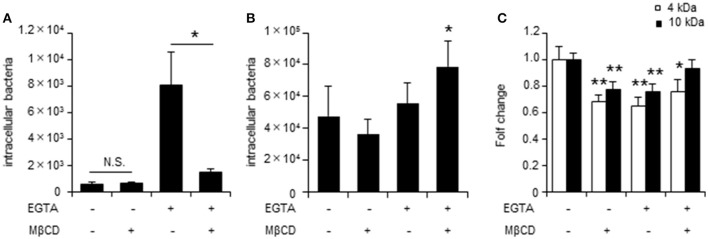
TJ disruption plays a key role in lipid raft-mediated *C. jejuni* invasion of polarized epithelial cells. The number of intracellular bacteria was measured by gentamycin protection assay. **(A)** Caco-2 cells were treated with 4 mM EGTA treatment for 20 min followed by 10 mM MβCD for 1 h and were infected with *C. jejuni* for 6 h. **(B)** Caco-2 cells pre-treated as in **(A)** were infected followed with *S*. Enteritidis for 1 h. **(C)** Caco-2 cells pre-treated as in **(A)** were incubated with 0.2 mg/ml 4 kDa (white bar) or 10 kDa FITC-dextran for 2 h. FITC-dextran uptake was evaluated by fluorescence measurements. Results are shown as the mean ± SD; *n* = 4–6. Significant difference from the control or EGTA treated group are shown: N.S.; not significant; ^*^P < 0.05; ^**^P < 0.01.

To further assess the role of TJs during *C. jejuni* invasion of polarized epithelial cells, invasion assays were performed using the Ca^2+^ chelator EGTA to disrupt TJs. EGTA treatment was associated with lower TER values and altered localization of the TJ marker protein ZO-1 (Supplementary Figure 2). EGTA treatment promoted *C. jejuni* invasion, which was significantly decreased by MβCD treatment only in cells with disrupted TJs. (Figure 3A). Similar results were observed for T-84 cells and Caco-2 cells following U18666A treatment (Supplementary Figure 2). To determine whether MβCD-mediated suppression of bacterial invasion in the presence of disrupted TJs was specific to *C. jejuni*, we also evaluated invasion of another human enteric pathogen, *S*. Enteritidis in polarized cells with disrupted TJs. In contrast to *C. jejuni* infection, EGTA treatment did not promote *S*. Enteritidis invasion and MβCD treatment did not decrease *S*. Enteritidis invasion in polarized cells with disrupted TJs (Figure 3B). Furthermore, we analyzed how EGTA or MβCD treatment affected endocytosis function by measuring uptake of FITC-dextran. EGTA or MβCD treatment had different effects on FITC-dextran uptake than that for *C. jejuni* invasion (Figure 3C). These results suggested that *C. jejuni* invasion has greater dependence on intact TJs in polarized cells than endocytosis.

### TJ formation affected *C. jejuni* invasion via lipid raft-mediated pathways in polarized epithelial cells

**Figure 4:**
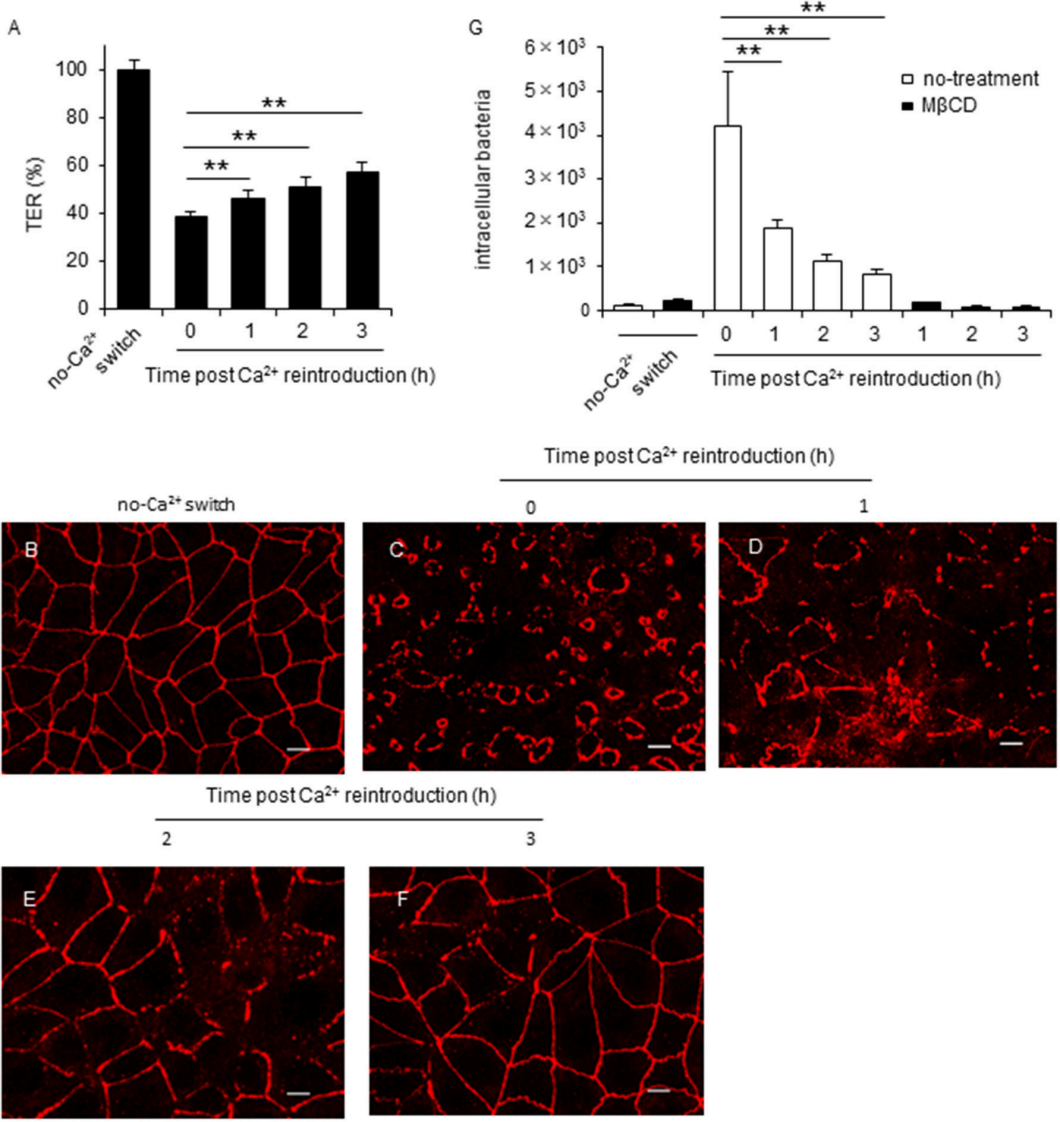
TJ disruption is tightly associated with lipid raft-mediated *C. jejuni* invasion in polarized epithelial cells. **(A–F)** Caco-2 cells cultured for 7 days on transwells and treated with 4 mM EGTA for 20 min. Following EGTA treatment, cells were incubated in normal culture medium. During the incubation, the TER value **(A)** and ZO-1 localization **(B–F)** were evaluated every hour for 3 h. TER values were calculated as the percentage of the TER value for untreated cells. Scale bar = 10 μm. **(G)** Caco-2 cells treated with 4 mM EGTA for 20 min were incubated in normal culture medium for the indicated time. After incubation, cells were pretreated with 10 mM MβCD for 1 h and infected with *C. jejuni* for 6 h. The number of intracellular bacteria was measured using a gentamycin protection assay. Results are shown as the mean ± SD; *n* = 4. Significant difference from the post Ca^2+^ reintroduction 0 h group are shown: N.S.; not significant; ^*^P < 0.05; ^**^P < 0.01.

Earlier studies reported that Ca^2+^ reintroduction recovered TER values and increased TJ integrity in EGTA-treated cells (Farshori and Kachar, 1999; Ronaghan et al., 2016). Therefore, we performed a calcium switch assay to examine the effect of TJ integrity on *C. jejuni* invasion. Consistent with earlier reports, we showed that Ca^2+^ reintroduction into Caco-2 cells restored the TER value (Figure 4A) and ZO-1 localization (Figures 4B–F) in a time-dependent manner. Furthermore, lipid raft-mediated *C. jejuni* invasion was significantly decreased following TJ restoration (Figure 4G). These data strongly suggested that cellular lateral or basolateral cell regions that are normally obscured by TJs are crucial for lipid raft-mediated *C. jejuni* invasion in polarized epithelial cells.

### *C. jejuni* invasive factor CapA is strongly associated with bacterial invasion in unpolarized cells

**Figure 5:**
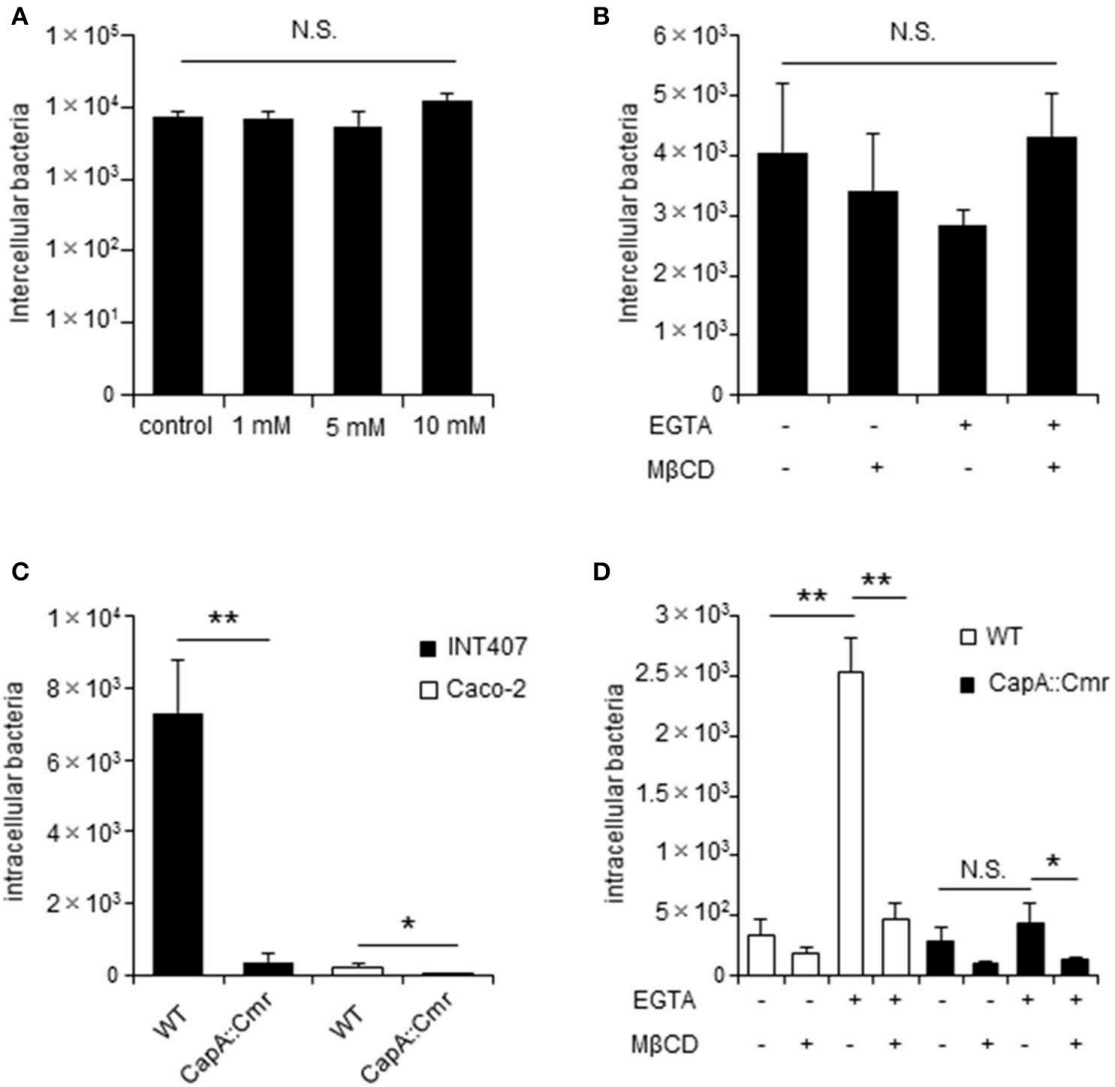
The *C. jejuni* invasive factor CapA is important for bacterial invasion of in TJs unformed cells. INT407 cells and Caco-2 cells were pretreated with the indicated compound and infected with *C. jejuni* for 1 or 6 h to evaluate bacterial adherence **(A,B)** and invasion **(D,E)**. **(A)** INT407 cells treated with 1–10 mM MβCD for 1 h. **(B)** Caco-2 cells pretreated with 4 mM EGTA for 20 min followed by treatment with 10 mM MβCD for 1 h. The number of intercellular bacteria was determined by an adhesion assay **(A,B)**. **(C)** INT407 cells (black bar) and Caco-2 cells (white bar) were infected with wild type (WT) *C. jejuni* or a CapA mutant strain. **(D)** Caco-2 cells pretreated with 4 mM EGTA for 20 min followed by 10 mM MβCD for 1 h and infected with WT *C. jejuni* (white bar) or a CapA mutant strain (black bar). The number of intracellular bacteria was measured by a gentamycin protection assay **(C,D)**. Results are shown as the mean ± SD; *n* = 4. Significant difference from the control group are shown: N.S.; not significant; ^*^P < 0.05; ^**^P < 0.01.

*Campylobacter jejuni* invasion proceeds in two steps: adhesion and invasion. However, which step TJs affect is unclear. Here we first checked whether EGTA and MβCD affected *C. jejuni* adhesion in non-polarized INT407 cells and polarized Caco-2 cells and found no effect on adhesion by the respective compounds (Figures 5A,B). As mentioned above, during the *C. jejuni* invasion step, MβCD significantly decreased *C. jejuni* invasion of polarized cells only in EGTA-treated cells (Figure 3A). To further confirm the interaction between *C. jejuni* invasion and TJs, we used invasion-defective *C. jejuni* strains. A *C. jejuni* deletion mutant lacking the autotransporter protein CapA (Ashgar et al., 2007) had significantly lower invasion into host cells, particularly non-polarized cells (Figure 5C). In contrast to WT *C. jejuni*, invasion of the CapA mutant did not increase after EGTA treatment of Caco-2 cells to disrupt TJs (Figure 5D). These results suggested that TJs do not affect bacterial adhesion, but instead are involved in the invasion step. Moreover, an invasive factor such as CapA might have an important role in *C. jejuni* invasion dynamics mediated by TJs in polarized cells.

### Intestinal inflammation promotes *C. jejuni* invasion in polarized epithelial cells

**Figure 6:**
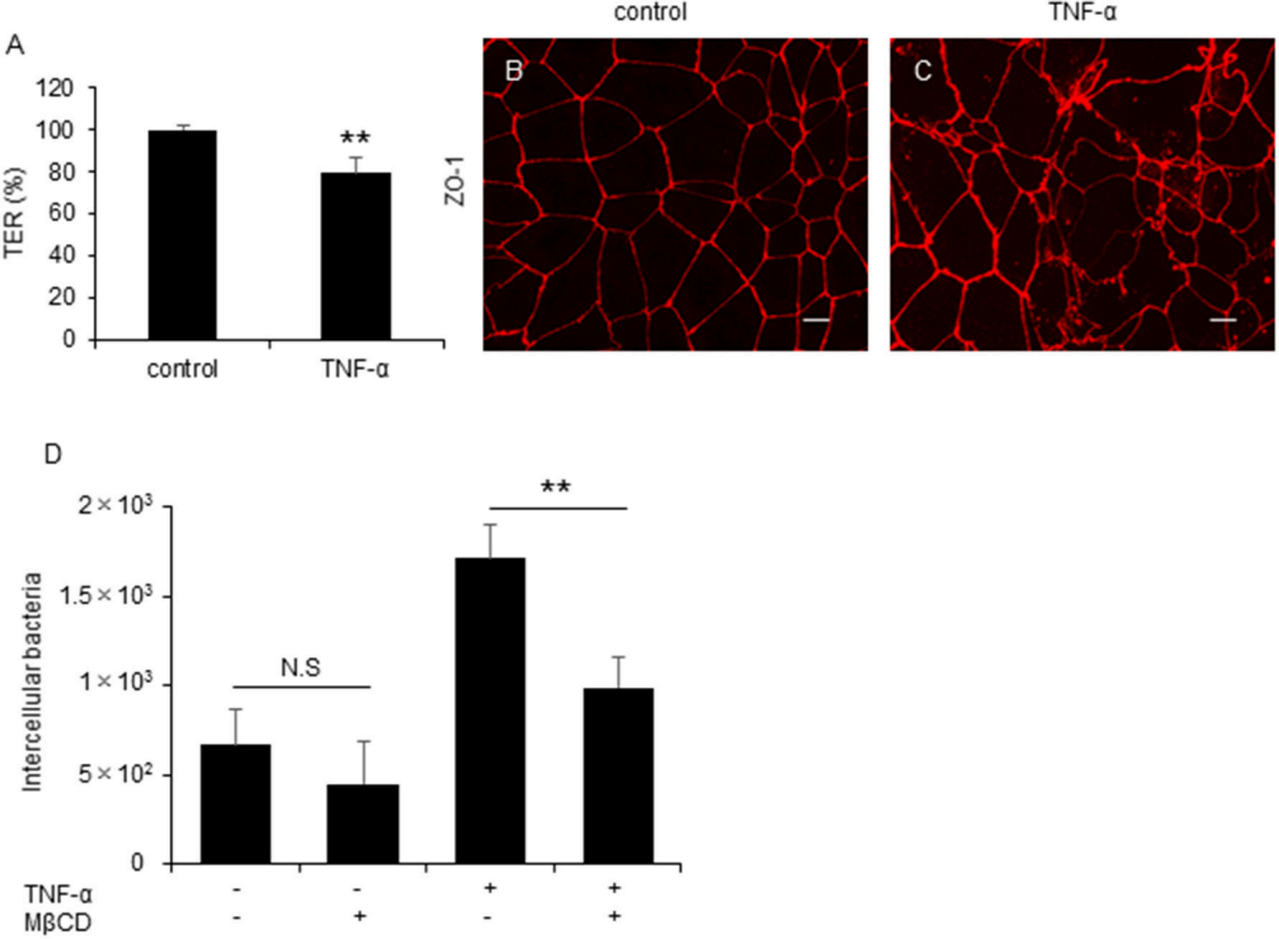
*C. jejuni* invasion is increased by active inflammation in polarized epithelial cells. **(A–C)** Caco-2 cells were treated with 20 ng/ml TNF-α for 48 h. Following TNF-α treatment, TER values **(A)** and ZO-1 localization **(B,C)** were evaluated. Scale bar = 10 μm. **(D)** Caco-2 cells were treated with 20 ng/ml TNF-α followed by treatment with 10 mM MβCD for 1 h and were infected with *C. jejuni* for 6 h. The number of intracellular bacteria was measured by gentamycin protection assay. Results are shown as the mean ± SD *n* = 4. Significant difference from the control or EGTA treated group are shown: N.S.; not significant; ^**^P < 0.01.

Several previous studies reported that active inflammation disrupts TJ formation and promotes barrier disruption in the intestine (Antoni et al., 2014; Lechuga and Ivanov, 2017) and proinflammatory cytokine, Tumor necrosis factor-α (TNF-α) play an important role in the intestinal barrier disruption (Ma et al., 2004). To investigate the influence of inflammation-mediated TJ disruption in *C. jejuni* invasion, we performed an invasion assay with polarized Caco-2 cells treated with TNF-α. Treatment of TNF-α significantly decreased TER value (Figure 6A) and induced ZO-1 localization change Figures 6B,C. Additionally, *C. jejuni* invasion was significantly increased and attenuated followed by MβCD treatment in TNF-α-treated polarized epithelial cells as measured by a gentamycin protection assay (Figure 6D). This result indicated that intestinal inflammation can induce barrier disruption and promote *C. jejuni* invasion in polarized epithelial cells.

## Discussion

*Campylobacter jejuni* invasion mechanisms have been studied using non-polarized epithelial cells. In this study, we examined whether TJs could prevent *C. jejuni* invasion by comparing *C. jejuni* invasion of polarized and non-polarized cells under a variety of treatments to disrupt TJs. Treatment with MβCD, a potent lipid raft-mediated endocytosis inhibitor that removes cellular cholesterol, decreased *C. jejuni* invasion in non-polarized INT407 cells, but invasion was not affected in polarized Caco-2 cells treated with MβCD (Figures 1A,D). However, in Caco-2 cells, removal of lipid rafts decreased *C. jejuni* invasion in TJ unformed or disrupted cells (Figures 2B, 3A). These data strongly suggest that TJ formation prevents *C. jejuni* entry in the lateral part of polarized epithelial cells. On the other hand, a CapA deletion mutant that lacks invasion activity (Ashgar et al., 2007) had decreased invasion into non-polarized epithelial cells and polarized epithelial cells with disrupted TJs (Figures 5C,D). These results suggested that bacterial adhesion or host endocytic uptake function was not related to bacterial invasion of TJ disrupted cells, and that bacterial invasive factors (e.g., CapA) participated in *C. jejuni* invasion of the lateral regions of TJ disrupted cells. In the final experiment, we investigated the impact of inflammatory cytokine-mediated on TJ suppression in *C. jejuni* invasion. Similarly, we found that TNF-α treatment increased *C. jejuni* invasion and the increases of invasion were suppressed by MβCD treatment (Figure 6D). This result indicated that TJ disruption together with severe inflammation promoted *C. jejuni* invasion in intestinal epithelial cells.

The intestinal epithelium provides a physical barrier against pathogenic bacteria, and the function of this barrier is mainly regulated by TJs (Schneeberger and Lynch, 2004; Suzuki, 2013). On the other hand, *C. jejuni* infection could induce TJ disruption mediated by proteases contained in bacterial outer membrane vesicles in polarized epithelial cells (Elmi et al., 2016). Thus, barrier dysfunction induced by *C. jejuni* infection could promote bacterial invasion into host cells, and additionally, bacterial invasion could cause severe inflammation. Some previous report indicated that *C. jejuni* could induce various pro-inflammatory cytokine production such as IL-6, IL-8 and TNF-α (Hickey et al., 1999; Al-Salloom et al., 2003; Friis et al., 2009) and the pro-inflammatory cytokine on TJs had been investigated. IL-6 could increase TJ permeability by inducing expression of claudin-2, which is pore-forming claudins (Suzuki et al., 2011), and TNF-α is known to cause TJ disruption by decreasing TJ protein occluding and ZO-1 expression (Ma et al., 2004; He et al., 2012). Therefore, severe inflammation associated with *C. jejuni* infection could lead to barrier disruption and induce further bacterial invasion. Thus, TJ disruption with intestinal inflammation may contribute to a vicious cycle that greatly influences pathogenicity in *C. jejuni* infection. In addition, various factors could increase the risk of TJ disruption in the several intestinal diseases, including irritable bowel disease, celiac disease, and alcoholic river disease (van Elburg et al., 1993; Piche et al., 2009; Rao, 2009). Therefore, effects from underlying diseases that involve TJ disruption may contribute to *C. jejuni* infection.

Bacterial invasion is an important step to initiate infection. In general, there are two different routes for pathogen internalization: the transcellular route and the paracellular route. Pathogenic bacteria can enter at the apical surface of the cell before being subsequently endocytosed and trafficked via transcellular mechanisms (Kazmierczak et al., 2001). On the other hand, in the paracellular route, specialized pathogens can cross the epithelial barrier and pass between connected epithelial cells by breaking cell junctional complexes such as TJs (Balkovetz and Katz, 2003). Previous studies revealed that *C. jejuni* could invade host cells either via the transcellular or paracellular routes (Brás and Ketley, 1999; Boehm et al., 2012). Many studies reported that *C. jejuni* invasion by the transcellular route was associated with IL-8 production (Hickey et al., 1999; Watson and Galán, 2005). However, relative to the transcellular route, the detailed mechanism and role of the paracellular route in *C. jejuni* infection is less well understood. Our demonstration indicated that TJ disruption and paracellular mechanisms were subsequently linked to efficient endocytic *C. jejuni* entry, and that transcellular mechanisms involving the lateral cell membrane, both alone and combined, was important for a better understanding of *C. jejuni* invasion dynamics in polarized epithelial cells.

In this study, in order to analyze the role of TJs on *C. jejuni* invasion pathway, we utilized EGTA and TNF-α. However, these compounds also affected Adherens Junctions (AJs), another cell-cell junction complex, in polarized Caco-2 cells (Yi et al., 2009; Goyer et al., 2016). Therefore, further study may need to investigate contribution of AJs on the *C. jejuni* invasion in polarized epithelial cells. In addition, to examine how *C. jejuni* could entry into polarized epithelia cells with TJ disruption, we investigated lipid rafts-mediated mechanism by using MβCD and U18666A, that deplete plasma membrane cholesterol. Importantly, previous studies have shown that Caco-2 cells lacking cavolin-1 and caveolin-2, which lipid rafts do not contain caveolae (Bradbury et al., 1999). Thus, Caco-2 cells indicate special type of lipid rafts and *C. jejuni* can invade into Caco-2 cells via caveolae independent mechanism (Konkel et al., 2013). Interestingly, lipid rafts-mediated endocytosis inhibitor decreased *C. jejuni* invasion in TJ disrupted Caco-2 cells (Figure 3A, Supplementary Figure 2), and our data suggest that *C. jejuni* could invade into polarized epithelial cells not caveolae but lipid rafts-mediated pathway. Lipid rafts provide a critical role in the localization of various receptor proteins and control of the subsequent signaling pathway, such as integrin β1 or EGFR (Ringerike et al., 2002; Wang et al., 2010). Additionally, activation of these receptor proteins is strongly related to *C. jejuni* invasion (Boehm et al., 2011; Eucker and Konkel, 2012; Konkel et al., 2013). Therefore, we hypothesized that *C. jejuni* could invade into polarized epithelial cells with TJ disruption by utilizing but lipid rafts-related receptor protein and signaling cascade.

Early report indicated that intestinal brush borders, a special type of microdomain in apical parts of host intestinal epithelium, contain lipid rafts (Danielsen and Hansen, 2007). However, our results demonstrated that lipid rafts depletion did not influence *C. jejuni* invasion in presence of TJ formation in polarized epithelial cells (Figure 1G). Host intestinal epithelium is usually covered with mucus layer, and mucus layer is also considered as defensive line against luminal bacterial infection (McAuley et al., 2007). Therefore, we also hypothesized that host mucus layer might prevent *C. jejuni* attachment to lipid rafts in apical cell surface and could contribute to *C. jejuni* invasion process in polarized epithelial cells. Thus, the investigation of interaction between host physiological factors and *C. jejuni* invasion might be important to reveal invasion mechanism in polarized epithelial cells.

Although our study has apparently demonstrated the role of lipid rafts in *C. jejuni* invasion in TJ disrupted cells, we were unable to identify the specific receptor for *C. jejuni* invasion. Hence, the finding in our study are subject to several limitations. First, the concentration of these cholesterol depleting agents, MβCD and U18666A were decided based on previous reports (Chen et al., 1993; Field et al., 2008; Elmi et al., 2012; Konkel et al., 2013), may not specific deplete only lipid rafts cholesterol but also may affect cellular cholesterol contents. Thus, further substantiate evidences regarding *C. jejuni* invasion, future studies are required to identify the specific receptor of *C. jejuni* invasion on the lateral cell surface and confirm the relation without inhibitors system such as knockdown or specific targets neutralizing antibody. In addition, while gentamycin protection assay is a mainstay technique in bacterial pathogenesis, it is not without weakness as this method indirectly evaluate the number of intracellular bacteria and have some limitations (et al., 2012). Therefore, another valuable approach, such as fluorescence or electron microscopy (Friis et al., 2005), might be required to investigate more detail the dynamics of *C. jejuni* invasion.

In conclusion, our study indicates that cellular TJ formation strongly affects *C. jejuni* invasion dynamics in polarized epithelial cells. In addition, our data supports the view that maintenance of TJ integrity in physiological condition should be considered for the development of improved preventive and therapeutic approaches to treat *C. jejuni* infections.

## Author contributions

SH and TS designed this research. SH, SA, JK, AN, YS, YK, AYT, and SF performed experiments. SH, MN, TU, and KM analyzed the data. SH, TS, and AKT drafted the manuscript. All authors have read the manuscript and approved its submission.

### Conflict of interest statement

The authors declare that the research was conducted in the absence of any commercial or financial relationships that could be construed as a potential conflict of interest.
