# Supplementary material for: Cellular Tight Junctions Prevent Effective Campylobacter jejuni Invasion and Inflammatory Barrier Disruption Promoting Bacterial Invasion from Lateral Membrane in Polarized Intestinal Epithelial Cells
Source: Front Cell Infect Microbiol. 2018 Jan 30;8:15. doi: 10.3389/fcimb.2018.00015 (PMC5797580; doi:10.3389/fcimb.2018.00015)
Supplement: Supplementary file 1 [file Presentation1.pdf]

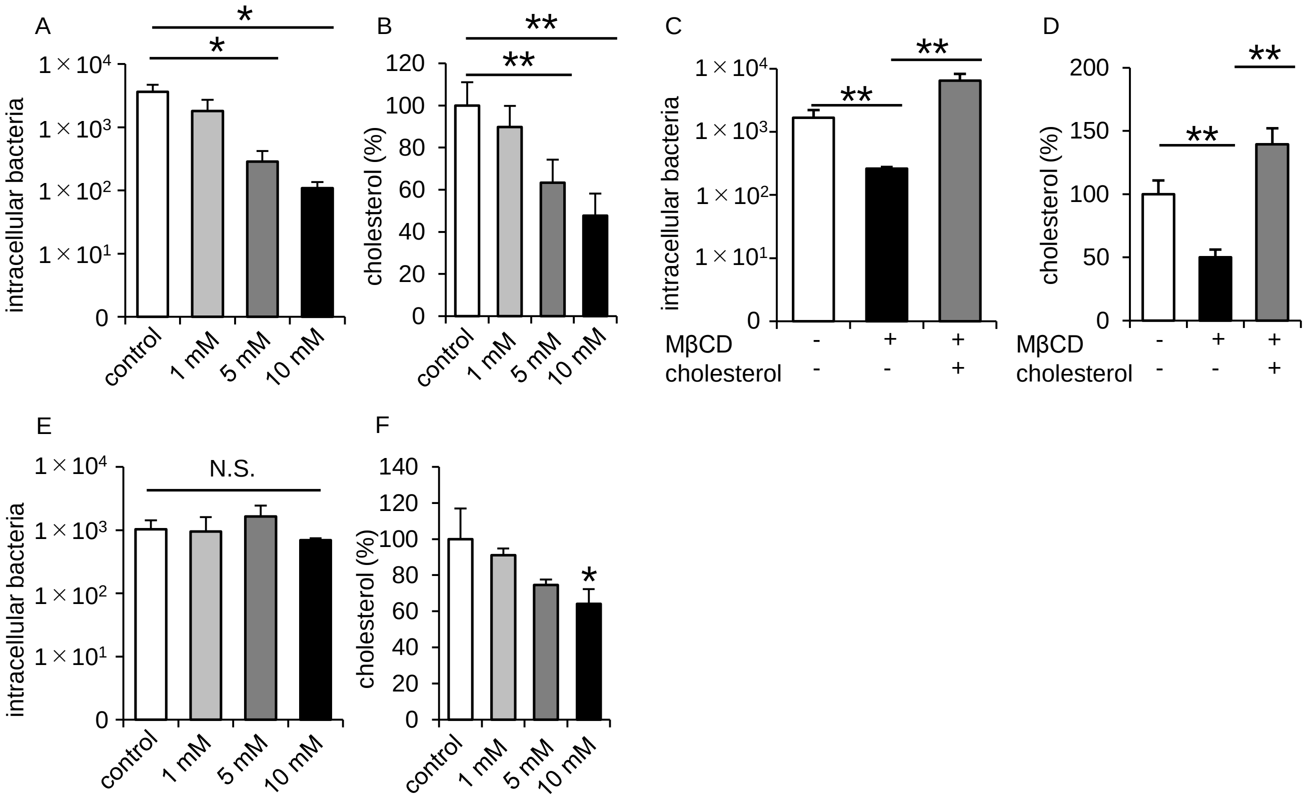

Supplemental Figure 1 | Non-polarized epithelial cells (HeLa cells) and polarized epithelial cells (T-84 cells) were pre-treated with MβCD and infected with *C. jejuni* for 6 h. The number of intracellular bacteria was measured by a gentamycin protection assay **(A, C, E)**. The level of intracellular cholesterol was measured using a cholesterol assay kit **(B, D, F)**. **(A, B)** HeLa cells pre-treated with 1-10 mM MβCD for 1 h. **(C, D)** HeLa cells pretreated with 10 mM MβCD for 1 h or MβCD-treated cells subsequently supplemented with 150 µg/µl cholesterol for 1 h prior to infection. **(E, F)** T84 cells pretreated with 1-10 mM MβCD for 1 h. Results are shown as the mean ± SD; n=4. Significant difference from the control or MβCD treated group are shown: N.S.; not significant; *, P <0.05; **, P <0.01.
