# Supplementary material for: Cellular Tight Junctions Prevent Effective Campylobacter jejuni Invasion and Inflammatory Barrier Disruption Promoting Bacterial Invasion from Lateral Membrane in Polarized Intestinal Epithelial Cells
Source: Front Cell Infect Microbiol. 2018 Jan 30;8:15. doi: 10.3389/fcimb.2018.00015 (PMC5797580; doi:10.3389/fcimb.2018.00015)
Supplement: Supplementary file 2 [file Presentation2.pdf]

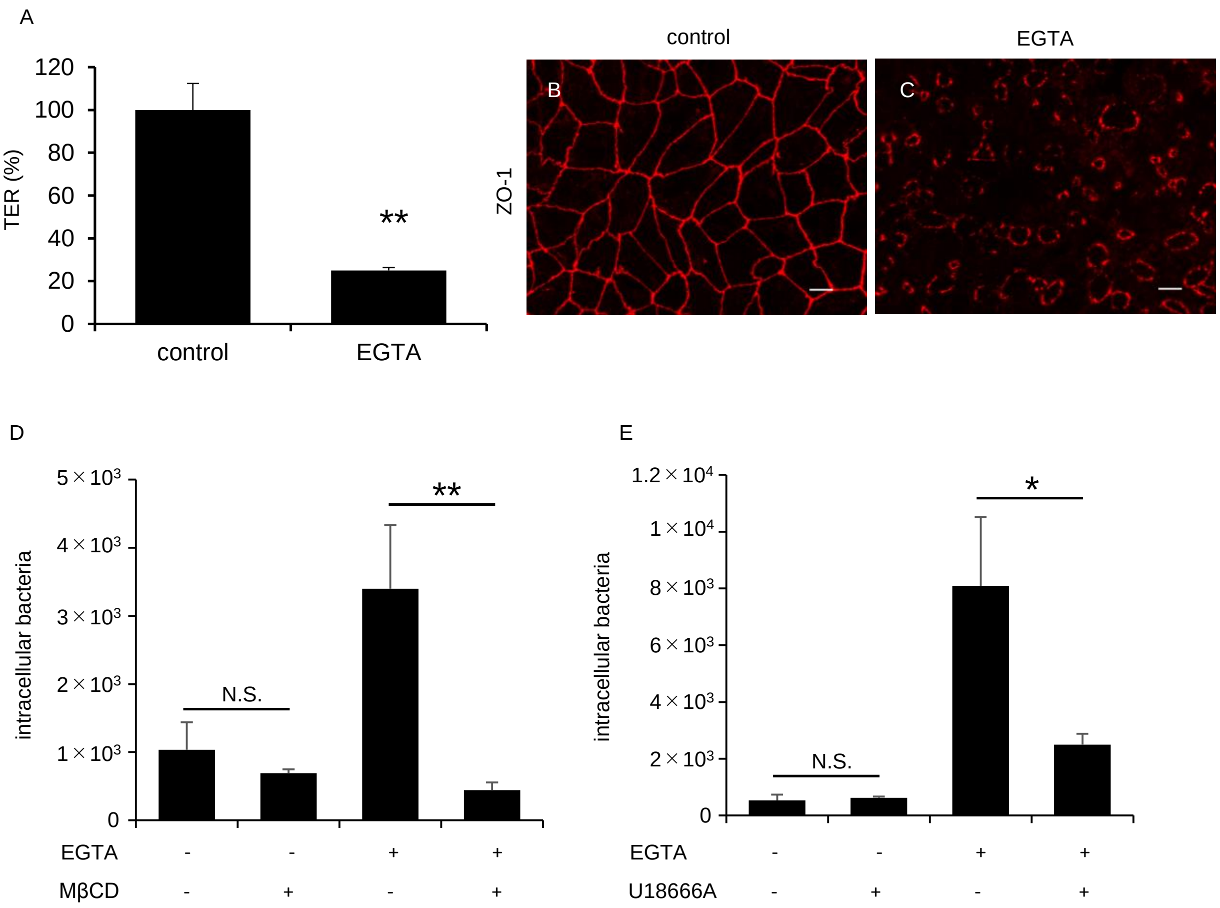

Supplementary Figure 2 | Lipid raft dysfunction reduced *C. jejuni* invasion of polarized epithelial cells with disrupted TJs. *A*, TER value measured in Caco-2 cells either left untreated or incubated with 4 mM, 20 min EGTA. The cells were then cultured on transwells. TER values were calculated as the percentage of TER values for untreated cells. *B*, Caco-2 cells cultured for 7 days treated with 4 mM EGTA for 20 min or left untreated and evaluated for ZO-1 localization (scale bar =10 μm). *C*, T-84 cells were treated with 4 mM EGTA treatment for 20 min followed by 10 mM MβCD for 1 h and were infected with *C. jejuni* for 6 h. *D*, Caco-2 cells were treated with 4 mM EGTA treatment for 20 min followed by 30 μM U18666A for 1 h and were infected with *C. jejuni* for 6 h. The number of intracellular bacteria was measured by a gentamycin protection assay (*C*, *D*). Results are shown as the mean ± SD; n=4. Significant difference from the control or MβCD treated group are shown: N.S.; not significant; *, P <0.05; **, P <0.01.
